# Molecular Cloning and Functional Analysis of *GmLACS2-3* Reveals Its Involvement in Cutin and Suberin Biosynthesis along with Abiotic Stress Tolerance

**DOI:** 10.3390/ijms22179175

**Published:** 2021-08-25

**Authors:** Asma Ayaz, Haodong Huang, Minglü Zheng, Wajid Zaman, Donghai Li, Saddam Saqib, Huayan Zhao, Shiyou Lü

**Affiliations:** 1 State Key Laboratory of Biocatalysis and Enzyme Engineering, School of Life Sciences, Hubei University, Wuhan 430062, China; asmaayaz@bs.qau.edu.pk (A.A.); haodonghuang@stu.hubu.edu.cn (H.H.); 202021107010975@stu.hubu.edu.cn (M.Z.); lidonghai17@mails.ucas.edu.cn (D.L.); huayanzhao@hubu.edu.cn (H.Z.); 2 Lushan Botanical Garden, Chinese Academy of Sciences, Nanchang 332900, China; shangla123@gmail.com; 3 State Key Laboratory of Systematic and Evolutionary Botany, Institute of Botany, Chinese Academy of Sciences, Beijing 100093, China; saddamsaqib.qau@gmail.com; 4 University of Chinese Academy of Sciences, Beijing 100049, China

**Keywords:** long-chain acyl-CoA synthetases, *Glycine max*, abiotic stresses, cutin, cuticular wax, suberin

## Abstract

Cutin and wax are the main precursors of the cuticle that covers the aerial parts of plants and provide protection against biotic and abiotic stresses. Long-chain acyl-CoA synthetases (*LACSs*) play diversified roles in the synthesis of cutin, wax, and triacylglycerol (TAG). Most of the information concerned with *LACS* functions is obtained from model plants, whereas the roles of *LACS* genes in *Glycine max* are less known. Here, we have identified 19 *LACS* genes in *Glycine max*, an important crop plant, and further focused our attention on 4 *LACS2* genes (named as *GmLACS2-1, 2, 3, 4*, respectively). These *GmLACS2* genes display different expression patterns in various organs and also show different responses to abiotic stresses, implying that these genes might play diversified functions during plant growth and against stresses. To further identify the role of *GmLACS2-3,* greatly induced by abiotic stresses, we transformed a construct containing its full length of coding sequence into *Arabidopsis*. The expression of *GmLACS2-3* in an *Arabidopsis atlacs2* mutant greatly suppressed its phenotype, suggesting it plays conserved roles with that of *AtLACS2*. The overexpression of *GmLACS2-3* in wild-type plants significantly increased the amounts of cutin and suberin but had little effect on wax amounts, indicating the specific role of *GmLACS2-3* in the synthesis of cutin and suberin. In addition, these *GmLACS2-3* overexpressing plants showed enhanced drought tolerance. Taken together, our study deepens our understanding of the functions of *LACS* genes in different plants and also provides a clue for cultivating crops with strong drought resistance.

## 1. Introduction

Cuticle covers the upper parts of land plants, preventing epidermal cells from water loss, chemicals, UV (ultraviolet) and irradiation and, thus, keeps plants surviving under extreme conditions [1,2]. Cuticle also plays a role during plant growth. Some papers have reported that defective cuticle causes organ fusion and flower sterility [3,4,5]. In addition, a recent study showed that cuticle is also deposited on the surface of root cap and lateral root primordia, where it provides protection and also participates in the formation of lateral roots [6]. Cuticle is composed of cutin and cuticular waxes. Cutin contributes to cuticle structure, whereas cuticular wax is deposited in the interior, which covers or fills the cutin matrix as intracuticular waxes or coats the outer surface of the epidermis in the form of an epicuticular film or crystals [2,7].

Common cutin monomers found in the literature are hexadecenoic acid, octadecenoic acids, diacids, and unsaturated fatty acids [2,8,9]. Cutin monomer synthesis initially starts in plastid where long-chain fatty acid (LCFA) de novo synthesis takes place, generating C16 and C18 acids. The fatty acid synthase (FAS) complex is involved in this process. The generated acids in the plastid are then transported to the endoplasmic reticulum (ER) and further modified. Many enzymes involved in the modification steps have been identified. *LACS1/2/4,* as the members of the Acyl-coenzyme A synthetase (ACS) family, are supposed to be responsible for attaching CoA to LCFA during cutin synthesis, providing a precursor for subsequent reactions [9]. Several members of the CYP86A family are reported to be involved in the terminal carbon reactions [2]. A member of the CYP77A family is involved in midchain reaction [10]. Three enzymes, i.e., *glycerol-3-phosphate acyltransferase 4* (*GPAT4*), *GPAT6,* and *GPAT8* facilitate cutin synthesis by transferring the acyl group from acyl-CoA to the glycerol-3-phosphate encoding acyltransferases [9,11]. However, some steps of cutin synthesis are still obscure, and the enzymes involved in these steps still need to be characterized. The wax synthesis also goes through two processes. The first process also occurs in the plastid where LCFAs are generated, and the second process takes place in the ER as well, which is related to the elongation and modification of LCFAs. In the ER, the LCFAs are converted into very long-chain fatty acids (VLCFAs) of C24–C36 chains by fatty acid elongase. Then, the VLCFAs are activated and modified into wax final constituents via two ways. One is the acyl reduction pathway generating primary alcohols and esters, and the other is the decarbonylation pathway producing aldehydes, alkanes, secondary alcohols, and ketones [7].

*LACS* facilitates the attachment of coenzyme-A to LCFAs (i.e.,12–20 carbons) and provides substrates for the synthesis of cutin and wax, the functions of which are well characterized in *Arabidopsis*. In total, nine *AtLACS* genes present in *Arabidopsis* are localized in different compartments and facilitate different pathways of lipid metabolism [12], among which *AtLACS1* and *AtLACS2* play a major role in the synthesis of wax and cutin [13]. *AtLACS1* is located in the ER and its mutation greatly reduced the wax amounts [13]. *AtLACS2* is also an ER-localized gene but plays an important role in cutin synthesis, and its loss of activity resulted in a significant reduction in cutin levels, resulting in lower membrane permeability and altered plant responses to abiotic and biotic stressors [13,14]. Moreover, it was also identified to be involved in suberin synthesis since the disruption of *AtLACS2* activity drastically reduced suberin amounts [15]. Suberin is mainly derived from long-chain aliphatic acids (>C16), the components of which are somewhat similar to cutin, thus partly sharing a biosynthesis pathway [9]. However, its distribution pattern is completely different from that of cutin, which is mainly deposited in root endodermis, wound sites, and abscission regions in *Arabidopsis* [9]. Apart from *Arabidopsis*, the functions of *LACS2* genes are also identified in other plants such as *Brassica napus*, *Helianthus annuus*, and *Malus domestica* [16,17,18,19]. Most of these genes displayed similar functions to *Arabidopsis* orthologs [16].

*G. max* is an essential oil crop, yielding about 30% of the consumable oil on the planet [20]. To cultivate crop plants with strong resistance will be helpful for improving crop yields. *LACS* genes play important roles in cuticle synthesis and are, thus, ideal targets for modifying crop properties. Till now, the function of *LACS* genes in *G. max* has scarcely been reported. Here, we proceeded with a thorough genome-wide evaluation of the *LACS* gene family in *G. max* and identified 19 *GmLACS*, among which there are four orthologs of *AtLACS2* genes (named as *GmLACS2-1, 2, 3, 4*). We further checked their tissue-specific expression pattern through real-time quantitative PCR (qRT-PCR). To find out if these *GmLACS2* genes are related to stress resistance, we also checked the responses of these genes to different chemicals causing abiotic stresses and finally focused on the *GmLACS2-3* gene, which was greatly induced by all treatments. We made a construct containing the full length of the *GmLACS2-3* coding sequence and then transferred it into an *atlacs2* mutant and a wild-type plant, respectively. We finally determined the cutin, suberin, and wax components in these transformed plants and evaluated the drought resistance of these plants; we found that *GmLACS2-3* is specifically involved in the synthesis of cutin and suberin but not related to wax synthesis, suggesting that the four *GmLACS2* genes’ functions have diversified during evolution. This study provides us more information on *LACS2* genes in crop plants and also gives directions for genetically modifying crop properties in the future.

## 2. Results

### 2.1. Identification and Homology Analysis of GmLACSs

**Figure 1:**
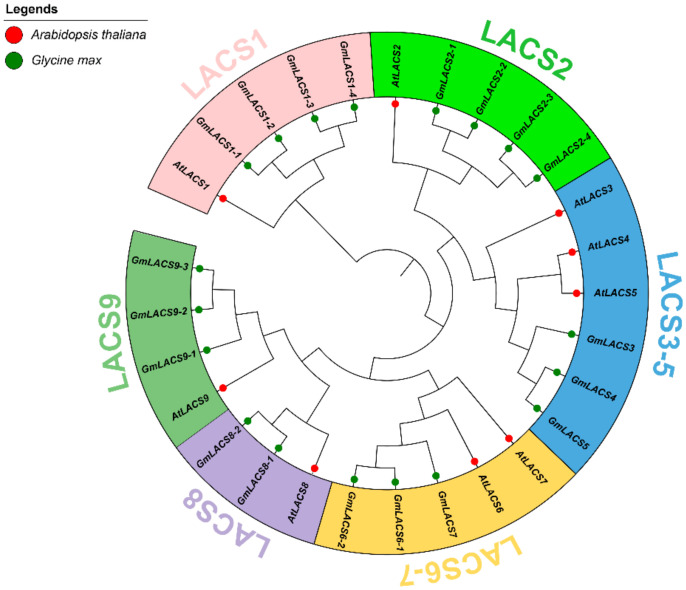
Maximum likelihood phylogenetic tree of the *LACS* gene family from *Glycine max* and *Arabidopsis thaliana*.

A total of 19 *LACSs* were identified from *G. max* based on their homologies to the nine known *LACS* from *Arabidopsis.* According to the classification criteria used for *Arabidopsis* and other plant species in previous studies [12,15], the *LACS*s were grouped into 6 clades with well-supported bootstrap values. *GmLACS* genes were placed in each clade with respective *Arabidopsis LACS* (Figure 1). Each clade contained a different number of *GmLACS*s, i.e., four *GmLACS*s each were grouped with *LACS1* and *2*. Three *GmLACS* genes were clustered with *AtLACS3, 4, 5*, while three *GmLACS* were grouped with *AtLACS6* and *7*. Two *GmLACS* shared the same clade with *AtLACS8*, while three *GmLACS* were grouped with *AtLACS9* (Figure 1). Phylogenetic analysis established six *AtLACS*/*GmLACS* sister pairs by more than 95% bootstrap value. Our analysis showed different branches distributing *Arabidopsis* and *G. max* homologs in functional clades. Phylogenetic results also revealed that proteins with higher homology might have the same functions and characteristics. All *LACS*s were renamed based on their orthologs from *Arabidopsis*. The putative proteins of *GmLACS* ranged from 625 (G_max_15G220900) to 725 (G_max_13G010100) amino acids in length. The molecular weights (MWs) ranged from 6.97 (G_max_15G220900) to 7.96 KDa (G_max_13G010100), theoretical isoelectric points (*pI*) ranged from 5.5 (G_max_15G220900) to 8.24 (G_max_06G112900), and the grand average of hydropathicity (GRAVY) ranged from −0.231 (G_max_19G218300) to 0.016 (G_max_14G149700). The average AA length, MW, *pI*, and GRAVY were 670, 7.46, 6.64, and −0.14, respectively. Complete information on the studied genes is provided in Table S2.

### 2.2. Expression Patterns of GmLACS2 Genes in Various Parts of G. max

**Figure 2:**
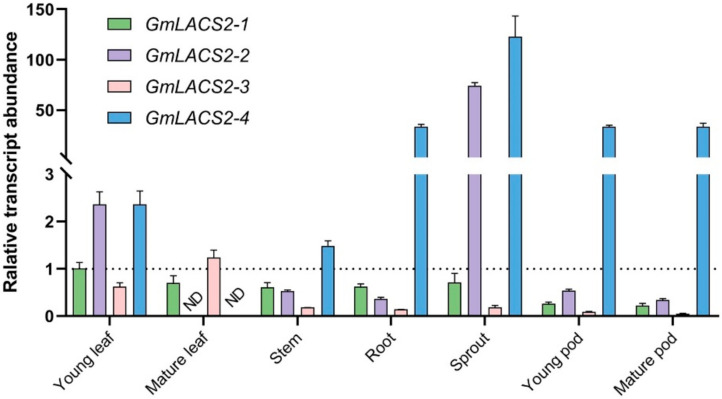
The expression patterns of different *GmLACS2* transcripts in different parts of *Glycine max*.

As compared with other *Arabidopsis LACS*s, *AtLACS2* plays the key role in cutin synthesis, and its mutation has a big effect on leaf permeability [13,14]. Thus, we focused our attention on its soybean orthologs—*GmLACS2-1*, *GmLACS2-2*, *GmLACS2-3*, and *GmLACS2-4*. The spatial expression pattern of the *GmLACS2*s was determined in different organs (Figure 2). *GmLACS2-1* was predominantly expressed in mature and young leaves; moderately expressed in sprout, stem, and root; and lowly expressed in young pod and mature pod. *GmLACS2-2* is highly expressed in sprouts and young leaves. Its transcript is also expressed in the stem, root, and young and mature pods. The expression pattern of *GmLACS2-3* was significantly higher in mature leaf compared to other transcripts. Moreover, *GmLACS2-3* showed slight expression in young leaf, followed by root and sprout, and stem showed no expression in the mature pod. Expression pattern analysis detected that *GmLACS2-4* is extremely expressed in the root, sprout, young pod, and mature pod. *GmLACS2-4* was significantly expressed in young leaf followed by the stem, whereas no transcripts were detected in mature leaf. These results reveal that these *GmLACS2* genes display different expression patterns in various organs, implying they might play different roles during plant development.

### 2.3. Expression of GmLACS2s under Different Stress Treatments

**Figure 3:**
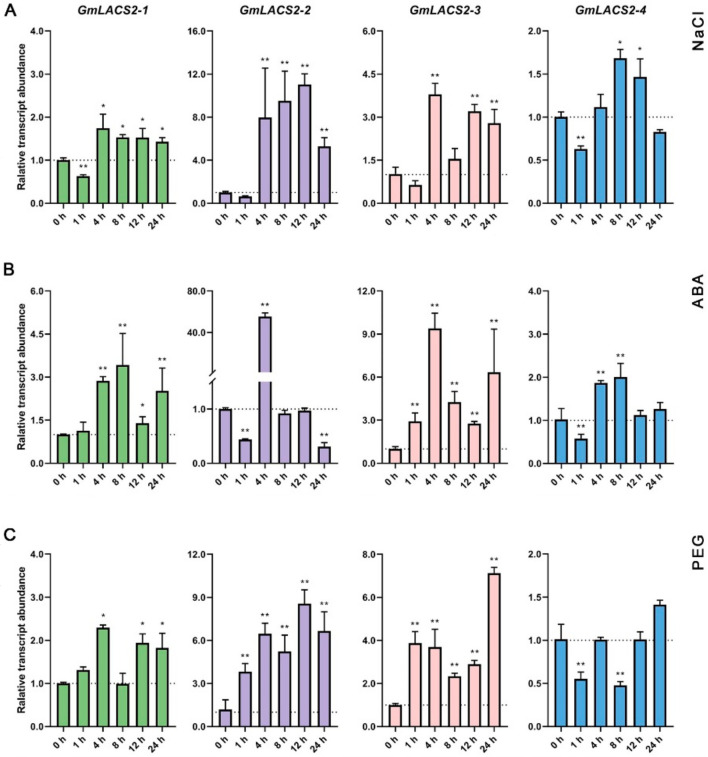
The expression profile of different *GmLACS2* transcripts under NaCl (**A**) ABA. (**B**) PEG. (**C**) treatments at different time intervals. * and ** respectively indicate significant difference (*p* < 0.05) and highly significant difference (*p* < 0.01).

Drought stress has been identified to induce cuticle biosynthesis [21]. To find out which *GmLACS2* genes might be involved in cuticle synthesis, we checked the responses of these genes to several chemicals triggering drought stress, including sodium chloride (NaCl), polyethylene glycol (PEG), and abscisic acid (ABA). These genes display different responses to the chemical treatments (Figure 3). *GmLACS2-1* was not induced by NaCl, slightly induced by PEG, and strongly induced by ABA, revealing that the *GmLACS2-1* gene displays a specific response to ABA treatment. However, its paralog, *GmLACS2-2*, displayed different expression patterns. It is significantly induced by each chemical; moreover, the effects of NaCl and PEG on its expression is maintained for a long time. Similar to *GmLACS2-2*, the expression of *GmLACS2-3* was also greatly induced by all chemicals (Figure 3). Moreover, *GmLACS2-3* expression under these treatments displays a biomodel pattern; the expression levels of this gene first peaked at 4 h after treatments, then gradually decreased, and increased again at a later stage. Different from *GmLACS2-3*, its paralog *GmLACS2-4* did not seem to be induced by all chemicals (Figure 3). Taken together, only *GmLACS2-2* and *GmLACS2-3* were strongly induced by all treatments, *GmLACS2-1* specifically responded to ABA treatment, and *GmLACS2-4* displayed little response, implying that these genes might play distinct roles under abiotic stresses.

### 2.4. GmLACS2-3 Localization at Subcellular Level

**Figure 4:**
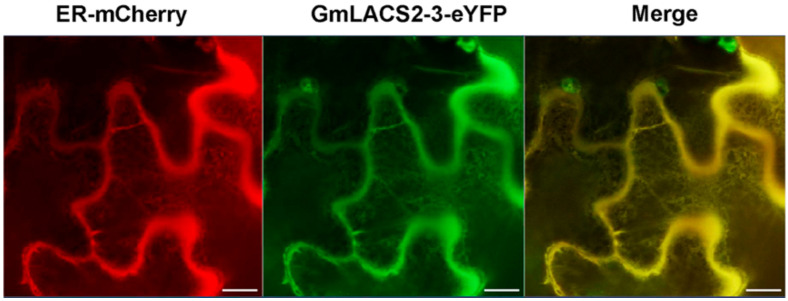
Subcellular localization of *GmLACS2-3* through ER-mCherry, *GmLACS2-3-eYFP,* and Merge fluorescent protein-tagged markers. Scale bars = 10 μm.

The subcellular localization of proteins is mainly associated with their biological function [22]. In higher plants, *LACS* proteins participate in various metabolic pathways and are localized in different compartments. In order to better understand the *GmLACS2* function in cuticle development, its subcellular localization was determined here. The *35S::GmLACS2-3::eYFP* construct was transiently co-expressed with the ER membrane marker CD3-959 in *Nicotiana benthamiana* leaves. As shown in Figure 4, the signals of *GmLACS2-3::eYFP* colocalized with CD3-959 as a reticulate network, the typical signal of ER protein, suggesting *GmLACS2-3* localizes to the ER. This expression pattern is consistent with that of many proteins involved in the synthesis of cutin and wax [2,7].

### 2.5. Restoration of Cutin-Deficient Phenotype of atlacs2 by GmLACS2-3

**Figure 5:**
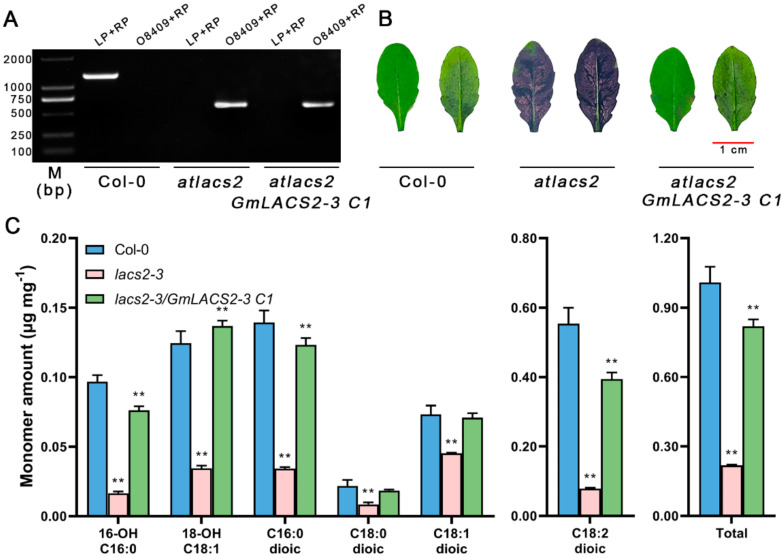
Complementation of cutin in transgenic lines. (**A**) Expression of *GmLACS2-3*. (**B**) Leaf visual appearance of wild-type (Col-0), mutant (*atlacs2*) and transgenic (*atlacs2/GmLACS*2-3 *C1*) lines. Scale bars = 1 cm. (**C**) Quantification of cutin monomers in the leaf of wild-type (Col-0), mutant (*atlacs2*) and transgenic (*atlacs2/GmLACS*2-3 *C1*) lines. ** represents highly significant difference (*p* < 0.01).

The *Arabidopsis atlacs2* mutant exhibits cutin monomer defects as well as increased cuticle permeability [13]. In order to verify whether the *GmLACS2-3* gene is also involved in the synthesis of cutin, we first overexpressed *GmLACS2-3* in the *atlacs2* mutant, and the RT-PCR results revealed *GmLACS2-3* was expressed in complementation lines (*atlacs2-3*/*GmLACS2-3 C1*) (Figure S1 and Figure 5A). To check cuticle permeability, we stained Col-0, *atlacs2,* and *atlacs2*/*GmLACS2-3 C1,* respectively, with TB staining solution. The *atlacs2* mutant showed dark staining due to cuticle defect, while the staining results of the *atlacs2*/*GmLACS2-3 C1* leaves were similar to that of Col-0 (Figure 5B), indicating that the expression of *GmLACS2-3* greatly suppressed the phenotype of *atlacs2*. To further confirm the staining results, we also determined the cutin contents in all plants. In *atlacs2,* the total amount of cutin was significantly decreased due to the simultaneous reduction of all cutin monomers (Figure 5C). However, in the complementation line of leaves, the amounts of all cutin monomers significantly increased, consistent with staining results. These results revealed that *GmLACS2* plays similar roles as *AtLACS2* in cutin synthesis and is also essential for maintaining cuticle integrity.

### 2.6. Overexpression of GmLACS2-3 in Arabidopsis Can Promote the Synthesis of Both Cutin and Suberin but Not Wax

**Figure 6:**
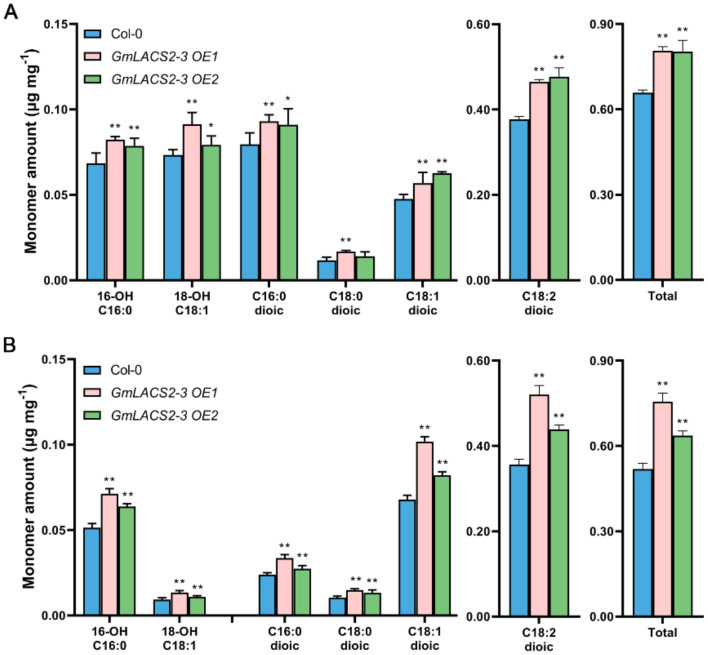
Cutin composition of leaf and stem of *Arabidopsis* wild-type Col-0, *GmLACS2-3 OE1*, and *GmLACS2-3 OE2*. The C16 and C18 labels on the *x*-axis represent the 16- and 18-carbon acid chains, respectively, whereas the number preceding “OH” indicates chain insertion point(s). Dioic represents dioic acid. The number of double bonds is indicated after the colon. Monomer amounts are expressed as µg·mg^−1^ leaf (**A**) and stem (**B**). The values shown are means ± SD (*n* = 4). * and ** respectively indicate significant difference (*p* < 0.05) and highly significant difference (*p* < 0.01).

To further assess the potential function of *GmLACS2-3*, we expressed it in *Arabidopsis* Col-0 and two transgenic lines, *GmLACS2-3 OE1* and *GmLACS2-3 OE2,* which were selected for further study. RT-PCR results showed that *GmLACS2-3* was highly expressed in both transgenic OE lines (Figure S1). Cutin monomers were quantified in stems and leaves of *Arabidopsis* wild-type Col-0 and the two OE lines, respectively. For leaves, each cutin monomer amount was increased in the OE line leaves, among which the major cutin monomer C18:2 dioic acid of *GmLACS2-3 OE1* and *GmLACS2-3 OE2* was increased by 23.36% and 26.53%, respectively. The rise of all cutin monomers finally resulted in the increased accumulation of total cutin monomer amounts. The total cutin monomer amounts of *GmLACS2-3 OE1* and *GmLACS2-3 OE2* were increased by 22.5% and 22.05%, respectively, compared to that of wild-type plants (Figure 6A). The stem cutin monomers of the OE lines displayed a similar variation tendency (Figure 6B). These results also confirmed that *GmLACS2-3* participates in cutin biosynthesis.

**Figure 7:**
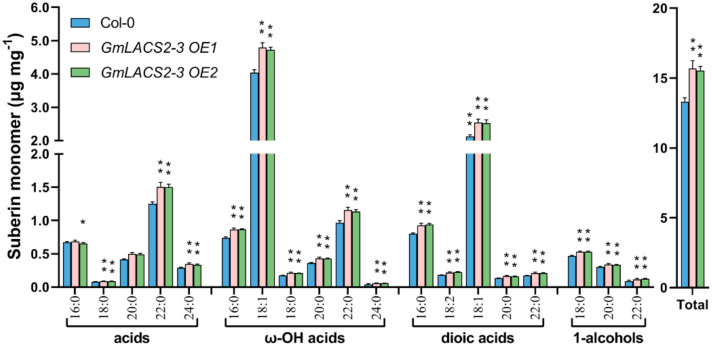
Suberin monomer composition in roots of the *Arabidopsis* wild-type Col-0, *GmLACS2-3 OE1,* and *GmLACS2-3 OE2*. The values shown are means ± SD (*n* = 4). * and ** respectively indicate significant difference (*p* < 0.05) and highly significant difference (*p* < 0.01).

Recently, we found that *AtLACS2* is also involved in suberin synthesis [15]. To determine whether *GmLACS2-3* plays a similar role in the synthesis of root suberin, we measured the suberin components of *GmLACS2-3 OE1*, *GmLACS2-3 OE2,* and Col-0 as well. Overall, the suberin contents of *GmLACS2-3 OE1* and *GmLACS2-3 OE2* were increased by 17.94% and 16.77%, respectively, compared to the Col-0 suberin level (Figure 7). The main suberin monomers of *Arabidopsis* include ω-OH acids, dioic acids, alcohols, and acids. The major suberin monomer C18:1 of ω-OH acids was increased by 18.68% and 17.02% in both *GmLACS2-3 OE1* and *GmLACS2-3 OE2* lines, respectively, over Col-0; C18:1 of dioic acids increased by 19.44% in *GmLACS2-3 OE1* and 18.8% in *GmLACS2-3 OE2* lines compared to Col-0. The results show that *GmLACS2-3* plays a role in suberin biosynthesis as well.

Since *LACS2* genes, such as *AtLACS2* and *MdLACS2* [13,17], are reported to play a role in the wax synthesis, we also checked the leaf and stem wax composition of *GmLACS2-3 OE1*, *GmLACS2-3 OE2,* and Col-0. However, no significant difference of either total wax amount or single wax constituent was found between Col-0 and the two *GmLACS2-3* OE lines (Figure S2 and Table S3). These results indicate that overexpression of *GmLACS2-3* in *Arabidopsis* has no significant effect on the synthesis of wax.

### 2.7. Effect of GmLACS2-3 Overexpression on Plant Response to Water Deficiency

**Figure 8:**
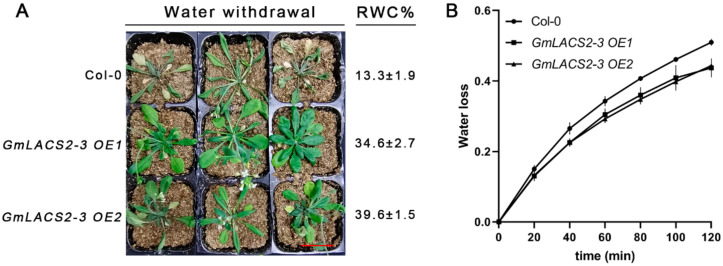
Wilting and drought resistance evaluation in *Arabidopsis* wild-type Col-0, *GmLACS2-3 OE1,* and *GmLACS2-3 OE2*. (**A**) The appearance of plants after 2 weeks withdrawal of water and RWC percentage. Scale bar = 3 cm. (**B**) Water loss rate of Col-0, *GmLACS2-3 OE1,* and *GmLACS2-3 OE2*. The values shown are means ± SD (*n* = 6), *p* < 0.05.

The 14-days old seedlings of Col-0 and the two *GmLACS2-3* OE lines were exposed to a 20-day water deficit condition. Dehydration treatments exerted different effects on these plants; most leaves of the two OE lines were turgid, whereas the leaves of Col-0 seriously wilted (Figure 8A). Meanwhile, we also determined the relative water content (RWC) of each plant; the RWCs of the two OE lines were higher than that for Col-0. Moreover, the transpiration rate was also assessed among these plants. The transpiration rate of the two OE lines was obviously lower than that for Col-0 (Figure 8). These results indicated that *GmLACS2-3* overexpression enhanced the cutin amounts, which finally resulted in enhanced drought resistance.

## 3. Discussion

Here, we identified 19 genes complementary to *LACS* in *G. max*; these were higher in number than *B. rapa* (12) and *B. oleracea* (16) and less than *B. napus* (34). However, *G. max* and *Brassica* species belong to different families; therefore, the number of genes varies in these species [23]. These differences indicate that the number of genes is based on genome size and gene duplication events in plants [24]. *GmLACS2* members in the same group exhibit relatively the same molecular masses and *pI* values, while different groups possess different *pIs* and molecular masses. These variations can lead to changes in gene structure and functions that play important roles in the gene family’s evolution. The extensive phylogenetic relationship showed 6 clades based on 28 genes of *Arabidopsis* and *G. max*. These results are similar to a previous study, which was based on *LACS* genes from 122 species [15]. In addition, expansion was observed in *GmLACS* genes. Specifically, *GmLACS1* and *GmLACS2* were more expanded, and each revealed 4 transcripts (Figure 1). These results indicated that both genes underwent a wide expansion in *G. max* compared to *Arabidopsis*. The phylogenetic tree shows that *GmLACS2* transcripts (*GmLACS2-1*, *GmLACS2-2*, *GmLACS2-3*, and *GmLACS2-4*) are very important for further evolutionary and functional evaluation. *GmLACS3*, *GmLACS4*, and *GmLACS5* were not expanded, while *GmLACS6-9* were less expanded relative to *GmLACS1* and *GmLACS2*. This phenomenon has been found in *Brassica* species, indicating that group duplication might result in the expansion of *LACS* genes [23].

Plant *LACS2* plays multiple roles in lipid metabolism, thus displaying diversified expression patterns. For example, in *Arabidopsis, AtLACS2* is involved in the biosynthesis of cutin and wax [13] and its transcripts are highly accumulated in elongating tissues [25]. A *Brassica napus* ortholog, *BnLACS2,* has been identified to participate in seed production, which is highly accumulated in developing seeds, where TAG is actively produced [18]. For an apple ortholog *MdLACS2*, it has been demonstrated to be involved in the synthesis of cuticular wax, which is highly expressed in pericarp [17]. Here, we examine the expression patterns of *GmLACS2s* in different organs and their responses to various stresses (Figure 2). *GmLACS2-3* has expression levels that are low in all tested organs compared with other genes. However, its transcripts are significantly induced by stress treatments (Figure 2 and Figure 3). A previous study showed that various stresses usually trigger the synthesis of cuticular wax and cutin [21]. Thus, we suspected that *GmLACS2-3* might participate in the biosynthesis of wax or cutin. Here, *GmLACS2-3* is identified to be involved in cutin synthesis (Figure 5 and Figure 6). Three genes besides *GmLACS2-3* also displayed distinct expression patterns in different organs or upon the stress treatments, implying they might have their specific functions during plant growth. Our future work will focus on identifying the functions of these genes. 

*GmLACS2* genes are involved in different pathways of lipid metabolism, including the synthesis of cutin, suberin, wax, and TAG, and the role of *AtLACS2* in these pathways has been identified [13,16,26]. Unlikely the *AtLACS2* gene in *Arabidopsis,* which possesses only one copy, there are four copies in soybean, and it seemed that these genes might own partial functions of *AtLACS2* in lipid synthesis. *GmLACS2-3* is a case. In this study, we have identified that *GmLACS2-3* is mainly responsible for the synthesis of cutin and suberin but plays no role in wax synthesis since its overexpression has little effect on wax contents (Figure S1). For *GmLACS2-4*, we supposed that it might be involved in the synthesis of TAG since its transcripts are abundantly accumulated in pods, where TAGs are usually synthesized and deposited. Moreover, a recent study revealed that the *BnLACS2* gene from *Brassica napus* is closely associated with TAG synthesis and highly expressed in seeds [18]. In addition, *GmLACS2-4* might also be associated with suberin synthesis since its transcripts are accumulated in root as well, where suberin is deposited. The functions of the other two *GmLACS2s* are still unknown, but we speculate that both genes might play different roles from *GmLACS2-3* and *GmLACS2-4* due to their distinct expression patterns. Taken together, *AtLACS2* exhibits broader roles within lipid synthesis pathways, while *GmLACS2* genes might have specific roles. Comprehensively investigating the roles of each *GmLACS2* gene will be helpful to expand our understanding of *GmLACS2* functions in lipid metabolism in crop plants.

Cutin is deposited on the surface of aerial parts together with wax, providing a protective layer against water loss [1,2,7], while suberin is scattered in root endodermis and also prevents the leakage of water and solutes from roots [27,28]. Therefore, both polymers are important for plant water retention capacity. Such is the case with *GmLACS2-3*. The expression of *GmLACS2-3* in *Arabidopsis* significantly increases the amounts of two polymers, implying that this gene is involved in both pathways. Moreover, its overexpression finally enhances plant drought resistance. A study that examined *MdLACS*2 isolated from apple also reported reduced epidermal permeability in *Arabidopsis,* which led to a decrease in water loss and promoted transgenic plant resistance to drought [17]. The prevention of water loss indicates that water loss may be well maintained due to the presence of cutin synthesis. Overall, our results indicate that *GmLACS2-3* is important for preventing water loss and, thus, a potential target gene for genetically modifying the drought resistance of crop plants in the future.

## 4. Materials and Methods

### 4.1. Identification and Phylogenetic Tree Reconstruction

To obtain putative *LACS* sequences from *G. max*, the previously reported and characterized *Arabidopsis* *LACS* sequences were acquired from “The Arabidopsis Information Resource” (TAIR) according to their Gene IDs. The *Arabidopsis* *LACS* protein sequences were used as probes to explore against *G. max* in the Phytozome database (www.phytozome.net, accessed on 23 January 2021.). The downloaded sequences were further screened using the methods of Ayaz et al. [15], and the phylogenetic tree was assembled utilizing FastTree v2.1.8 (https://journals.plos.org/plosone/article?id=10.1371/journal.pone.0009490, accessed on 30 July 2021) with default options [29] in the GenomeNet (www.genome.jp/, accessed on 23 January 2021). The validity of the mapped tree was checked out by a bootstrapping approach with 1000 replicates. For visualization and annotation of the tree, iTOL was utilized by default setting [30].

### 4.2. Plant Materials and Treatments

Seeds of Williams 82, the soybean cultivar used to produce the reference genome sequence, were sterilized with 10% sodium hypochlorite (NaOCl), rinsed 5 times with distilled water, and soaked overnight. The seedlings were grown in the greenhouse with a soil mixture of peat and vermiculite at a 1:1 ratio at 26 °C and 12/12 h (light/dark) under relative humidity up to 60%. Fifteen-day-old seedlings were subjected to various stress treatments after the expansion of the first trifoliate leaves. The roots were absorbed in PEG-simulated drought (15% PEG 6000), salinity (150 mM NaCl), and ABA (100 μM ABA) solutions for stress. The seedlings were incubated for various time intervals, i.e., 0, 1, 4, 8, 12, and 24 h. The trifoliate leaves were harvested at each time interval. The experiment was conducted in triplicate for each treatment. All samples were frozen in liquid nitrogen and stored in a −80 °C freezer for further analysis. For tissue-specific expression pattern experiments, soybean growth conditions were the same as in the above-mentioned conditions. Soybean organs at different stages were collected, frozen in liquid nitrogen, and then store in a −80 °C freezer for further experiments.

Seeds of *Arabidopsis* plants were sterilized through ethanol 75% (*v*/*v*) and NaOCl 10% (*v*/*v*) for 4 min. After that, the seeds were rinsed 5 times in distilled water to remove impurities. The purified seeds were then vernalized at 4 °C for 2–3 days and germinated on 1/2; MS medium containing 0.8% (*w*/*v*) agar and 0.5% (*w*/*v*) sucrose. After germination, the seedlings were moved to soil pots for cultivation. The temperature and light conditions were set at 22 ± 2 °C (16/8 h light/dark cycle). *lacs2-3* (GABI-Kat line 368C02) was kindly provided by Dr. Christiane Nawrath (University of Lausanne, Switzerland) [14]. To avoid confusion, *atlacs2* is used to reference *lacs2-3* throughout the paper.

### 4.3. RNA Extraction, cDNA Synthesis, and Quantitative Real-Time PCR Analysis

Triplicate biological samples of either soybean or *Arabidopsis* were used for the extraction of total RNA using the Trizol total RNA extraction kit (SIMGEN, Beijing, China) according to the manufacturer’s recommendations. Genomic DNA was removed using a DNase I. A nano photo spectrometer was used to check the quality and quantity of extracted RNA. Reverse transcription from each sample was performed. The first-strand cDNA was constructed using the PrimeScript First-Strand cDNA synthesis kit (Takara, Dalian, China), following the manufacturer’s recommendations. The specific primers for the expression levels of *GmLACS2s* were designed on the online server Snapgene (www.snapgene.com/, accessed on 4 February 2021.) (Table S1). Semi-quantitative RT-PCR and qRT-PCR were utilized to determine the transcript levels of *GmLACS2s* using *AtACTIN2* (AT3G18780) in *Arabidopsis* and *GmACTIN* (NM_001289231) in soybean as internal control, respectively. Triplicate biological qRT-PCR reactions were proceeded according to the manual (2× SuperReal PreMix Plus). Relative expression levels of target genes were calculated based on the 2^−ΔΔ*C*t^ method. The primers were designed for our selected genes with Primer3 primer (www.bioinfo.ut.ee/primer3-0.4.0/, accessed on 4 February 2021.) and assembled by TSINGKE Biological Technology (Wuhan, China) (Table S1).

### 4.4. Cloning and Production of Transgenic Plants

The *GmLACS2-3* open reading frame was amplified from the cDNA of *Glycine max* using gene-specific primers. The corresponding PCR products were cloned into a pFGC-eYFP vector. The gene-plasmid construct was transferred into an *Agrobacterium tumefaciens* GV3101 strain through the freeze–thaw method. Subsequently, the *Arabidopsis* plants (Col-0 and *atlacs2*) were transformed by the *Agrobacterium* strain through the floral-dipping method. For the complementation test, T0 transgenic seeds were harvested. After cold treatment for 3 days at 4 °C, the transgenic seeds were evenly sown in the soil. After germination, 1:1000 diluted Basta herbicide was sprayed on the plants to screen for positive transgenic lines. Finally, the confirmed OE lines (*GmLACS2-3 OE1*, and *GmLACS2-3 OE2*) and the *atlacs2-*complemented line (*GmLACS2-3 C1*) were selected for further study.

### 4.5. Subcellular Localization Determination

The subcellular localization assay proceeded with the *35S::GmLACS2-3::eYFP* construct, and protein colocalization was observed by the mCherry fluorescent protein-tagged ER marker CD3-959. Overnight bacterial cultures were centrifuged, followed by suspension in liquid Murashige and Skoog medium for tobacco infiltration analysis. Utilizing 1 mL plastic syringes, the obtained solution was infiltrated into *N. benthamiana* leaves. The infiltrated leaves were marked after infiltration, and the plants were transferred to the growth chamber [31]. The photographs were imaged by a ZEISS LSM 980 confocal microscope.

### 4.6. Cutin, Suberin, and Wax Evaluation

For the cutin analysis method, described by Lü, Song, Kosma, Parsons, Rowland and Jenks [13], we used the leaves and stems of 6-weeks old plants of Col-0, *GmLACS2-3 OE1*, *GmLACS2-3 OE2, atlacs2-3, and GmLACS2-3 C1*. Internal standard methyl heptadecanoate was used. The initial temperature of the column was set at 80 °C and raised at 15 °C min^−1^ up to 200 °C, then raised at 2 °C min^−1^ up to 280 °C. The waxes were evaluated according to methods described by Chen et al. [32]. Suberin composition from the roots of Col-0, *GmLACS2-3 OE1*, and *GmLACS2-3 OE2* was determined following the methods described by Ayaz et al. [15].

### 4.7. Drought and Cuticle Permeability Assay

All seeds, including Col-0, *GmLACS2-3 OE1,* and *GmLACS2-3 OE2,* were sown in biological triplicates, including 90 plants for each line after sterilization and vernalization at 4 °C for 3 days. After 2 weeks, we transferred the pots to a dry tray for wilting assays. Every two days, the pots were moved to counteract the effect of the pot’s position. For RWC measurements, the rosettes’ fresh weight (FW) of all triplicates was obtained through a microbalance. The turgid weights (TWs) were obtained as the rosettes were submerged in deionized distilled water for 6 h and then blotted dry. Rosettes were then dried at 80 °C in an oven and reweighted to determine the dry weight (DW). The rosettes’ RWC was measured as (FW − DW)/(TW − DW) × 100.

The TB staining method was followed, as described by Tanaka et al. [33]. About 4-week-old leaves of the Col-0, *atlacs2*, and *GmLACS2-3 OE* lines were immersed in 0.05% TB solution for 5 min and then rinsed with water. For water loss assessment, about 4-week-old plants were used to quantify the excised rosette. Prior to measurements, the plants were dark-acclimated for three hours. Entire rosettes were removed from the roots and soaked in water immediately in the dark for 60 min to equilibrate water content. After that, the extra water was removed from the leaves gravimetrically, and the weights were examined via a microbalance. The obtained data were represented as % of initial water-saturated fresh weights.

## 5. Conclusions

In this study, we identified 19 genes of *GmLACS* in the *G. max* genome. In addition, the phylogenetic tree, which was based on 28 *AtLACS/GmLACS* genes from *Arabidopsis* and *G. max*, resulted in VI clades with well-supported bootstrap values. qRT-PCR analysis showed that the *GmLACS2* genes exhibit distinct tissue-specific expression patterns and also showed different responses to NaCl, PEG, and ABA treatments. We further identified the function of *GmLACS2-3* since it is strongly induced by abiotic stresses. The expression of *GmLACS2-3* in *Arabidopsis* confirmed that this gene plays a key role in the synthesis of cutin and suberin but is not involved in wax synthesis. Moreover, *GmLACS2-3* plays an important role in controlling water loss by contributing to the biosynthesis of cutin and suberin. Overall, these results reveal that *GmLACS2-3* is a potential target for genetically modifying the qualities of crop plants.

## Data Availability

Data are available from the authors on request.

## Supplementary Materials

The following are available online at https://www.mdpi.com/article/10.3390/ijms22179175/s1. Figure S1: Expression of various transcripts of *GmLACS2-3* in Col-0, *GmLACS2-3 OE1*, *GmLACS2-3 OE2,* and *atlacs2/GmLACS2-3 C1* and *atlacs2*. Figure S2: Wax composition in leaf and stem of *Arabidopsis* wild-type Col-0, *GmLACS2-3 OE1* and *GmLACS2-3 OE2*. Table S1: List of Primers used in this study, Table S2: The sequences characteristics of identified *LACSs* genes in *Glycine max*. Table S3: Cuticular wax composition of both inflorescence stems and leaves of *Arabidopsis* Col-0, *GmLACS2-3 OE1,* and *GmLACS2-3 OE2*. Values shown are means ± SD (μg dm^−2^), total wax amounts, and coverage of individual compound classes (*n* = 4). —, Undetectable.
