# Supplementary material for: Molecular Cloning and Functional Analysis of GmLACS2-3 Reveals Its Involvement in Cutin and Suberin Biosynthesis along with Abiotic Stress Tolerance
Source: Int J Mol Sci. 2021 Aug 25;22(17):9175. doi: 10.3390/ijms22179175 (PMC8430882; doi:10.3390/ijms22179175)
Supplement: Supplementary file 1 [file ijms-22-09175-s001.zip › ijms-1352904-supplementary.pdf]

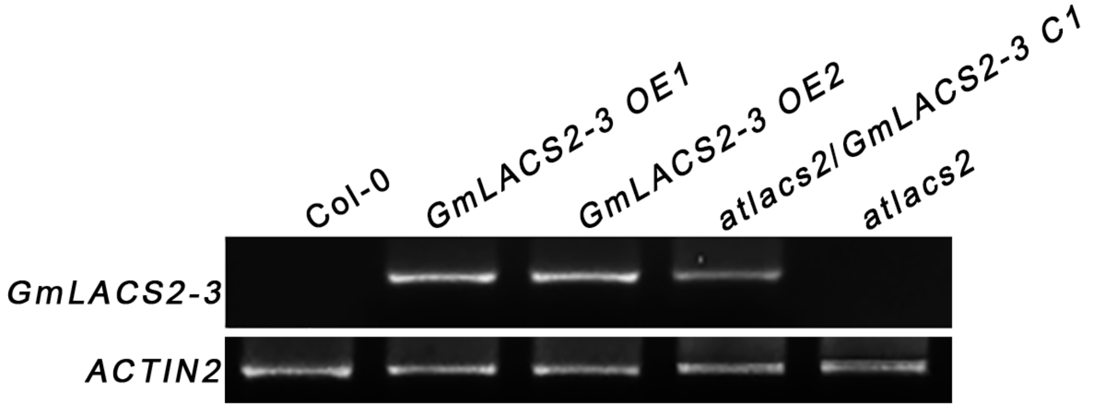

Figure S1. Expression of various transcripts of *GmLACS2-3* in Col-0, *GmLACS2-3 OE1*, *GmLACS2-3 OE2* and *atlacs2*/*GmLACS2-3 C1* and *atlacs2*.

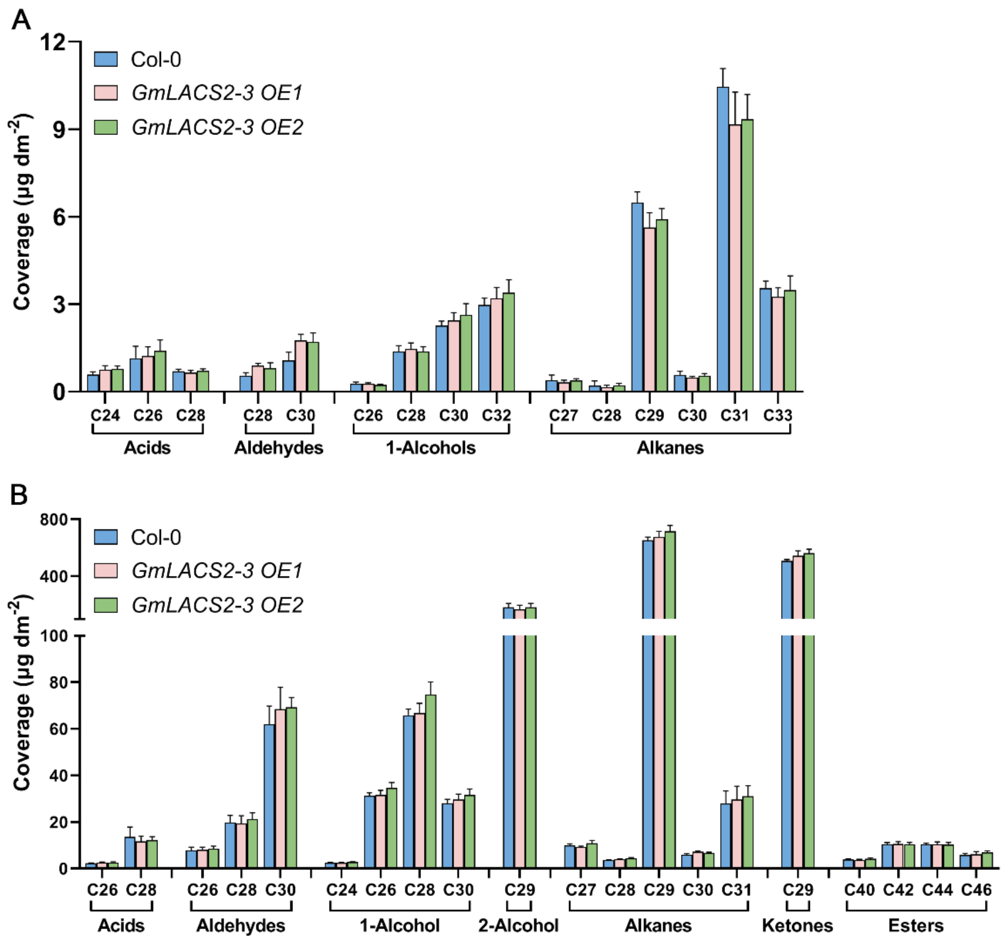

Figure S2. Wax composition of leaf and stem of *Arabidopsis* wild-type Col-0, *GmLACS2-3 OE1* and *GmLACS2-3 OE2*. (A) Wax profile in leaves of Col-0, *GmLACS2-3 OE1* and *GmLACS2-3 OE2*. (B) Wax profile in stems of Col-0, *GmLACS2-3 OE1* and *GmLACS2-3 OE2*. Wax coverage is expressed as wax amounts per leaf/stem surface area ($mg.dm^{-2}$). Each wax constituent is designated by carbon chain length and is labeled by chemical class along the x axis. Values shown are means ± SD (n=4).

Table S1. List of Primers used in this study.

**Name**	Sequence	Purpose
**GABI368C02-LP**	GAGGCTGTGAAGAAATATCCG	For genotyping lacs2-3 T-DNA insersion lines
**GABI368C02-RP**	GTACTCGAGACTTCCCCGAAG	For genotyping lacs2-3 T-DNA insersion lines
**o8409**	ATATTGACCATCATACTCATTGC	T-DNA left-border primer
**LACS2.3-F**	ACAATTACATTTACAATTACGATG CCAGAGGTTTATACTGT	Amplification of *GmLACS*2-3 (G.max 20G007900)
**LACS2.3-R**	AGCTCCTCGCCCTTGCCCATGAAT TGTGCTCTTTTGCCCAAT	Amplification of *GmLACS*2-3 (G.max 20G007900)
***ACTIN*-F**	CGGTGGTTCTATCTTGGCAT	Amplification of *GmACTIN*
***ACTIN*-R**	GTCTTTCGCTTCAATAACCCT	Amplification of *GmACTIN*
**QRT-F1**	GTTCGCCTGTTGTTCCACTT	qRT-PCR, *GmLACS*2-1 (G.max 12G047400)
**QRT-R1**	GCCATTCTCCAATGTCACCT	qRT-PCR, *GmLACS*2-1 (G.max 12G047400)
**QRT-F2**	TGCTTCTCGTGGGAAGAGTT	qRT-PCR, *GmLACS*2-2 (G.max 11G122500)
**QRT-R2**	GTTCGCCTGTTGTTCCACTT	qRT-PCR, *GmLACS*2-2 (G.max 11G122500)
**QRT-F3**	GGCTTTCATGGCTGAAGTGT	qRT-PCR, *GmLACS*2-3 (G.max 20G007900)
**QRT-R3**	CCTATTGATGAGCCCTTGGA	qRT-PCR, *GmLACS*2-3 (G.max 20G007900)
**QRT-F4**	TTGGATGAGCTCAACAGCAC	qRT-PCR, *GmLACS*2-4 (G.max 07G161900)
**QRT-R4**	CAAAGGGAATTGGTTCCAGA	qRT-PCR, *GmLACS*2-4 (G.max 07G161900)
**RT-F**	CTAGGTGGACGTGTTCGCAT	RT-PCR, *GmLACS*2-3 (G.max 20G007900)
**RT-R**	ACCACAACAGCCACCAAGAA	RT-PCR, *GmLACS*2-3 (G.max 20G007900)
***ACTIN*2-F**	GTTGGTGATGAAGCACAATCCAAG	RT-PCR (AT3G18780)
***ACTIN*2-R**	CTGGAACAAGACTTCTGGGCATCT	RT-PCR (AT3G18780)

Table S2. The sequences characteristics of identified *LACSs* genes in *Glycine max*.

S. No	Gene Id*	Molecular weight	Theoretical *pI*	AA length	Grand average of hydropathicity (GRAVY)
1.	G_max_01G225200	75365.95	6.88	671	-0.164
2.	G_max_02G010300	74382.04	6.54	660	-0.215
3.	G_max_03G221400	74409.94	6.78	662	-0.194
4.	G_max_05G216600	74670.93	6.10	665	-0.204
5.	G_max_06G112900	76017.50	8.24	694	-0.066
6.	G_max_07G161900	73852.74	6.20	660	-0.197
7.	G_max_10G010800	74516.20	6.73	660	-0.220
8.	G_max_10G249700	76880.03	7.86	696	-0.124
9.	G_max_11G017900	74031.44	7.76	663	-0.153
10.	G_max_11G122500	73788.63	6.23	656	-0.223
11.	G_max_12G047400	73843.48	5.58	656	-0.229
12.	G_max_13G010100	79550.53	7.83	725	-0.107
13.	G_max_13G079900	76131.58	5.91	696	-0.021
14.	G_max_14G149700	70974.48	5.90	647	0.016
15.	G_max_15G220900	69692.28	5.55	625	-0.034
16.	G_max_19G218300	74643.20	6.50	662	-0.231
17.	G_max_20G007900	73831.71	6.39	660	-0.208
18.	G_max_20G060300	74317.36	6.53	674	-0.092
19.	G_max_20G143900	76907.96	6.77	698	-0.134

***** G_max indicates Glyma., while number indicates the accession number.

Table S3. Cuticular wax composition of both inflorescence stems and leaves of *Arabidopsis* Col-0, *GmLACS2-3 OE1* and *GmLACS2-3 OE2*. Values shown are means ± SD (μg dm^{-2}), total wax amounts, and coverage of individual compound classes (n = 4). –, Undetectable.

**Sample**	Total	Fatty Acids	Aldehydes	1-Alcohols	2-Alcohol	Alkanes	Ketone	Esters
**Inflorescence stems**								
**Col-0**	1655±89	16.0±4.3	89.7±12.1	127.4±5.95	182±128	701±27	509±10	30.5±2.2
GmLACS2-3 OE1	1711±136	14.3±2.5	95.9±13.8	130.1±9	169±28	727±45	544±35	30.8±3.4
GmLACS2-3 OE2	1801±127	14.7±1.8	98.9±8.2	143.4±11	184±26	767±48	561±29	31.8±2.9
**Rosette leaves**								
**Col-0**	41.6±3.7	2.44±0.56	1.62±0.37	6.86±0.67	-	21.7±1.7	-	-
GmLACS2-3 OE1	42.1±5.0	2.61±0.53	2.64±0.30	7.35±0.89	-	19±2.1	-	-
GmLACS2-3 OE2	44.3±5.4	2.89±0.55	2.50±8.2	7.63±10.2	-	19.9±1.9	-	-
